# Author Correction: Macrophage-associated wound healing contributes to African green monkey SIV pathogenesis control

**DOI:** 10.1038/s41467-019-13816-9

**Published:** 2019-12-13

**Authors:** Fredrik Barrenas, Kevin Raehtz, Cuiling Xu, Lynn Law, Richard R. Green, Guido Silvestri, Steven E. Bosinger, Andrew Nishida, Qingsheng Li, Wuxun Lu, Jianshui Zhang, Matthew J. Thomas, Jean Chang, Elise Smith, Jeffrey M. Weiss, Reem A. Dawoud, George H. Richter, Anita Trichel, Dongzhu Ma, Xinxia Peng, Jan Komorowski, Cristian Apetrei, Ivona Pandrea, Michael Gale

**Affiliations:** 1 Department of Microbiology, University of Washington, Seattle, WA USA; 2 Department of Cell and Molecular Biology, Uppsala University, Uppsala, Sweden; 3 Division of Infectious Diseases, Department of Medicine, University of Pittsburgh, Pittsburgh, PA USA; 4 Department of Microbiology and Molecular Genetics, School of Medicine, University of Pittsburgh, Pittsburgh, PA USA; 5 Department of Pathology, School of Medicine, University of Pittsburgh, Pittsburgh, PA USA; 6 Department of Immunology, University of Washington, Seattle, WA USA; 7 Center for Innate Immunity and Immune Diseases, University of Washington, Seattle, WA USA; 8 Department of Pathology & Laboratory Medicine, Emory University, Atlanta, GA USA; 9 Division of Microbiology & Immunology, Yerkes National Primate Research Center, Emory University, Atlanta, GA USA; 10 Nebraska Center for Virology, School of Biological Sciences, University of Nebraska-Lincoln, Lincoln, NE USA; 11 Washington National Primate Research Center, University of Washington, Seattle, WA USA; 12 Divison of Laboratory Animal Resources, University of Pittsburgh, Pittsburgh, PA USA; 13 Department of Orthopedic Surgery, University of Pittsburgh, Pittsburgh, PA USA; 14 Department of Molecular Biomedical Sciences, North Carolina State University, Raleigh, NC USA; 15 Institute of Computer Science, PAN, Warsaw Poland

**Keywords:** Data integration, Functional clustering, Viral infection, Systems biology

Correction to: *Nature Communications* 10.1038/s41467-019-12987-9, published online 8 November 2019.

The affiliation of Jan Komorowski with the Institute of Computer Science, PAN, Warsaw, Poland, was inadvertently omitted in the original version of this Article. This has now been corrected in both the PDF and HTML versions of the Article.

