# Supplementary material for: Macrophage-associated wound healing contributes to African green monkey SIV pathogenesis control
Source: Nat Commun. 2019 Nov 8;10:5101. doi: 10.1038/s41467-019-12987-9 (PMC6841668; doi:10.1038/s41467-019-12987-9)
Supplement: Supplementary file 1 — Supplementary Information [file 41467_2019_12987_MOESM1_ESM.pdf]

Supplementary figure 1

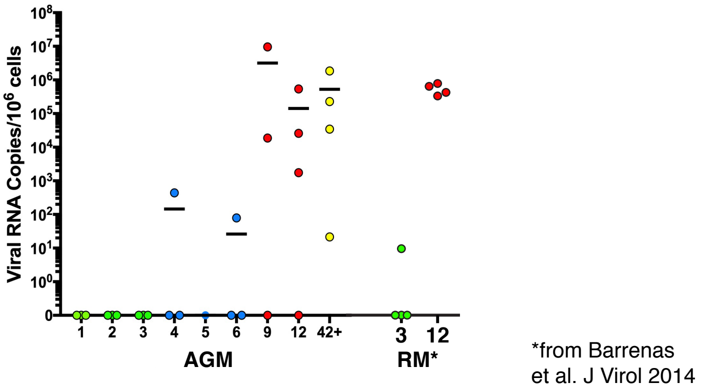

Supplementary figure 2

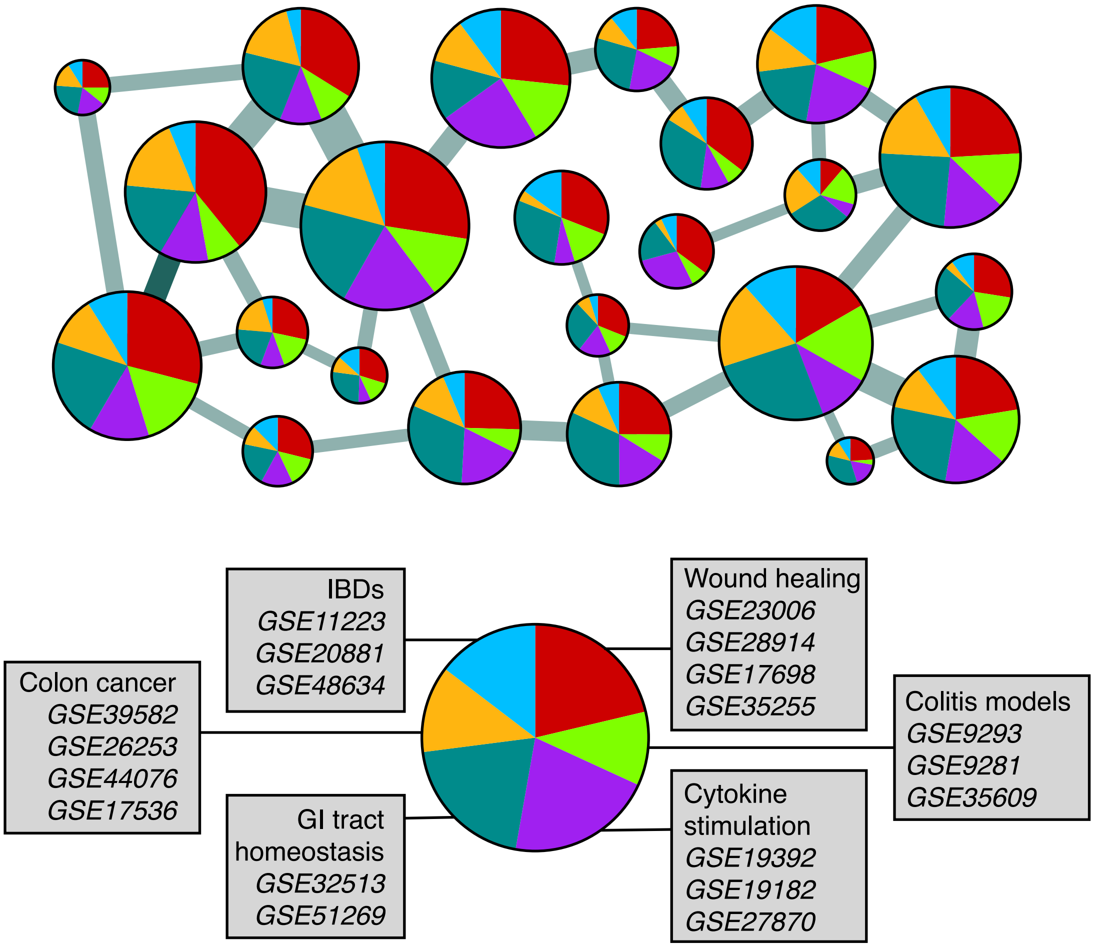

Supplementary figure 1. Viral load in rectal tissues from AGM and RM (RM data previously published in Barrenas et al. JVirol 2014)

Supplementary figure 2. Contribution of reference datasets to each module. For each interaction in the network, we determined the reference dataset that showed the highest correlation coefficient. For each module, we then determined the number of interactions that were most strongly supported by each dataset type (wound healing, colitis models, cytokine stimulation, GI tract homeostasis, colon cancer, inflammatory bowel diseases.

Supplementary figure 3

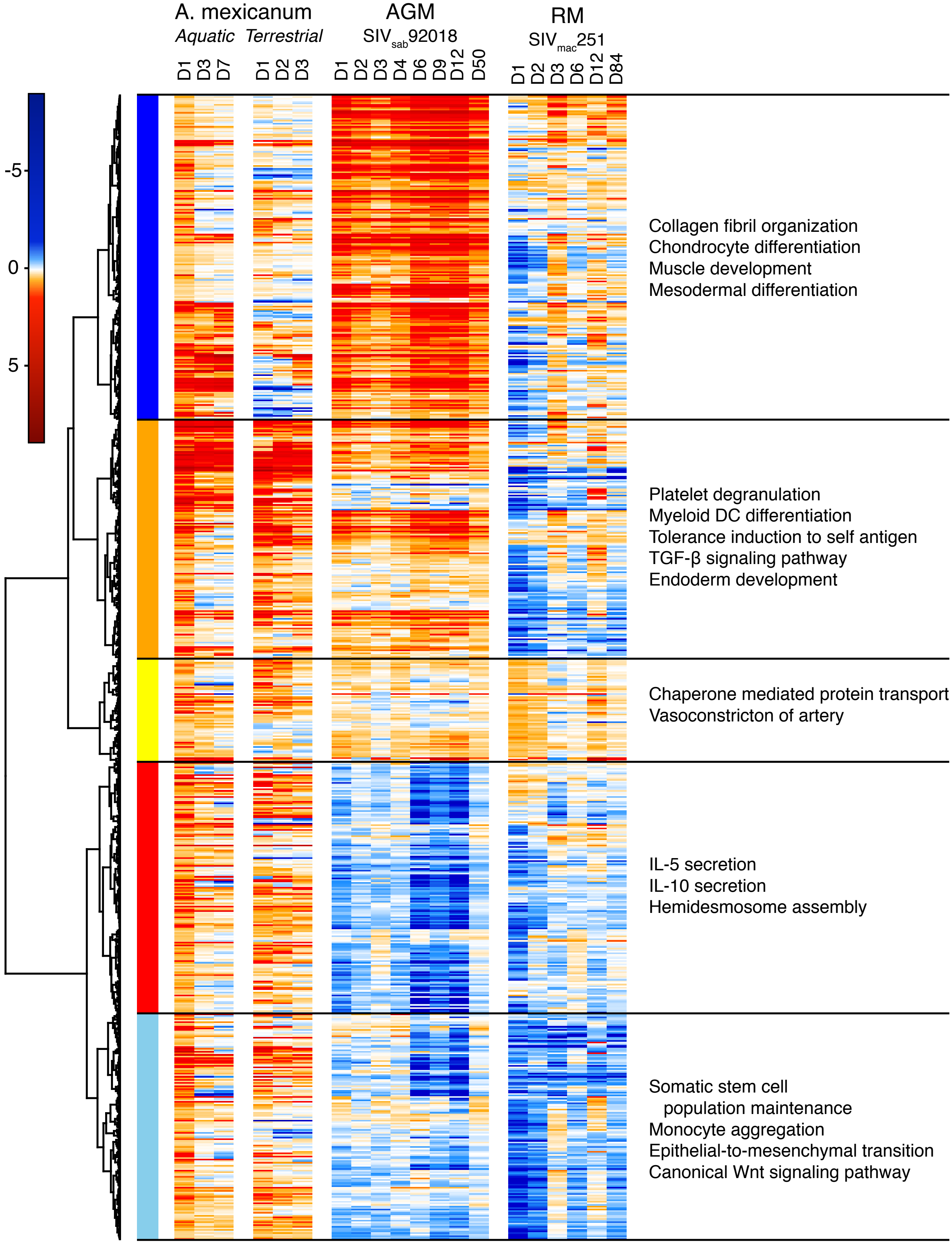

Supplementary figure 3. Heatmap showing all up regulated genes from day 1 of aquatic axolotl wound repair. Columns include wound repair in aquatic and terrestrial (mammal-like) axolotls and acute SIV infection in AGMs and RMs.

Supplementary figure 4 - AGM Baseline

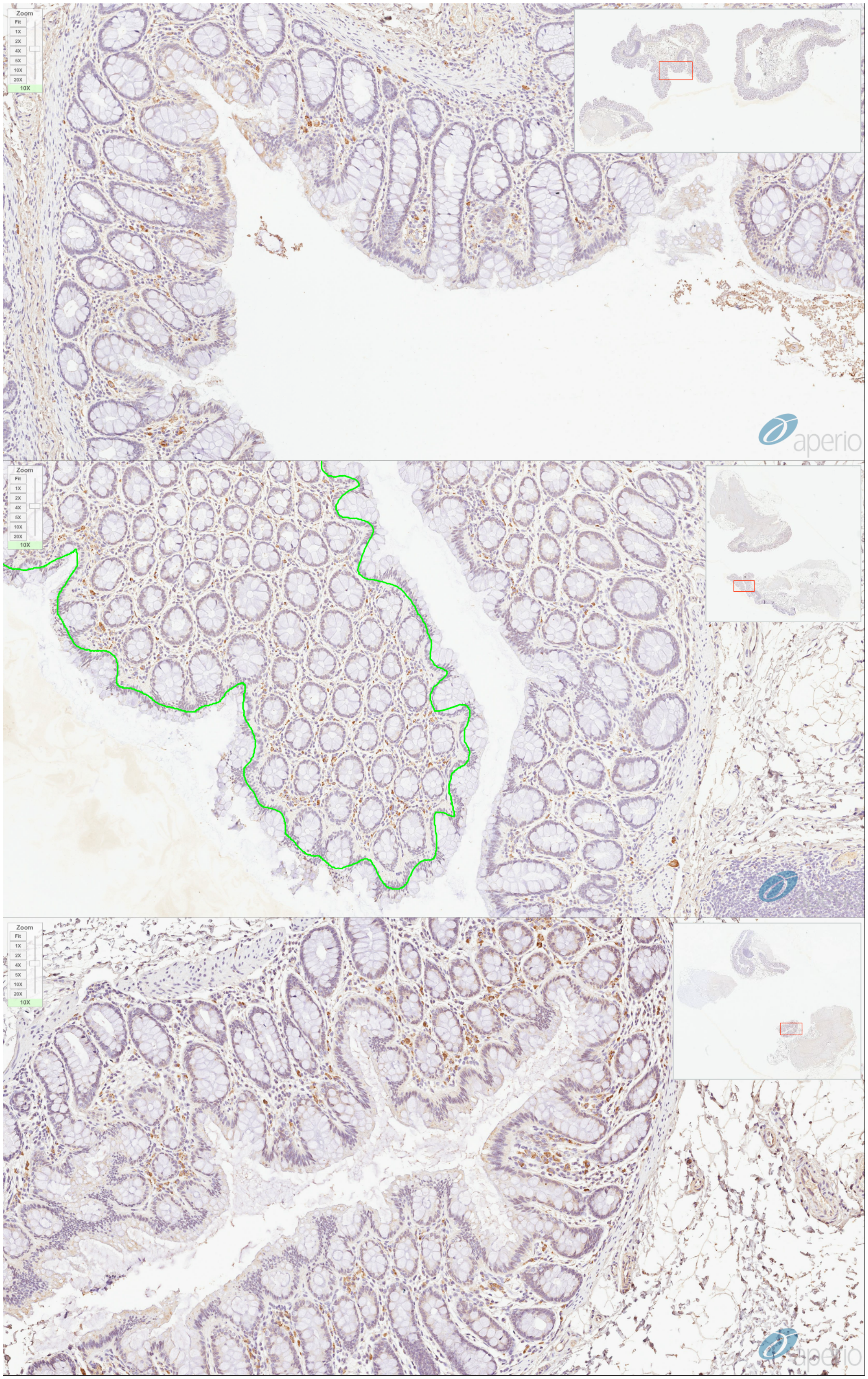

Supplementary figure 4. Immunohistochemistry of fibronectin in SIV uninfected AGMs.

Supplementary figure 5 - AGM Day 1

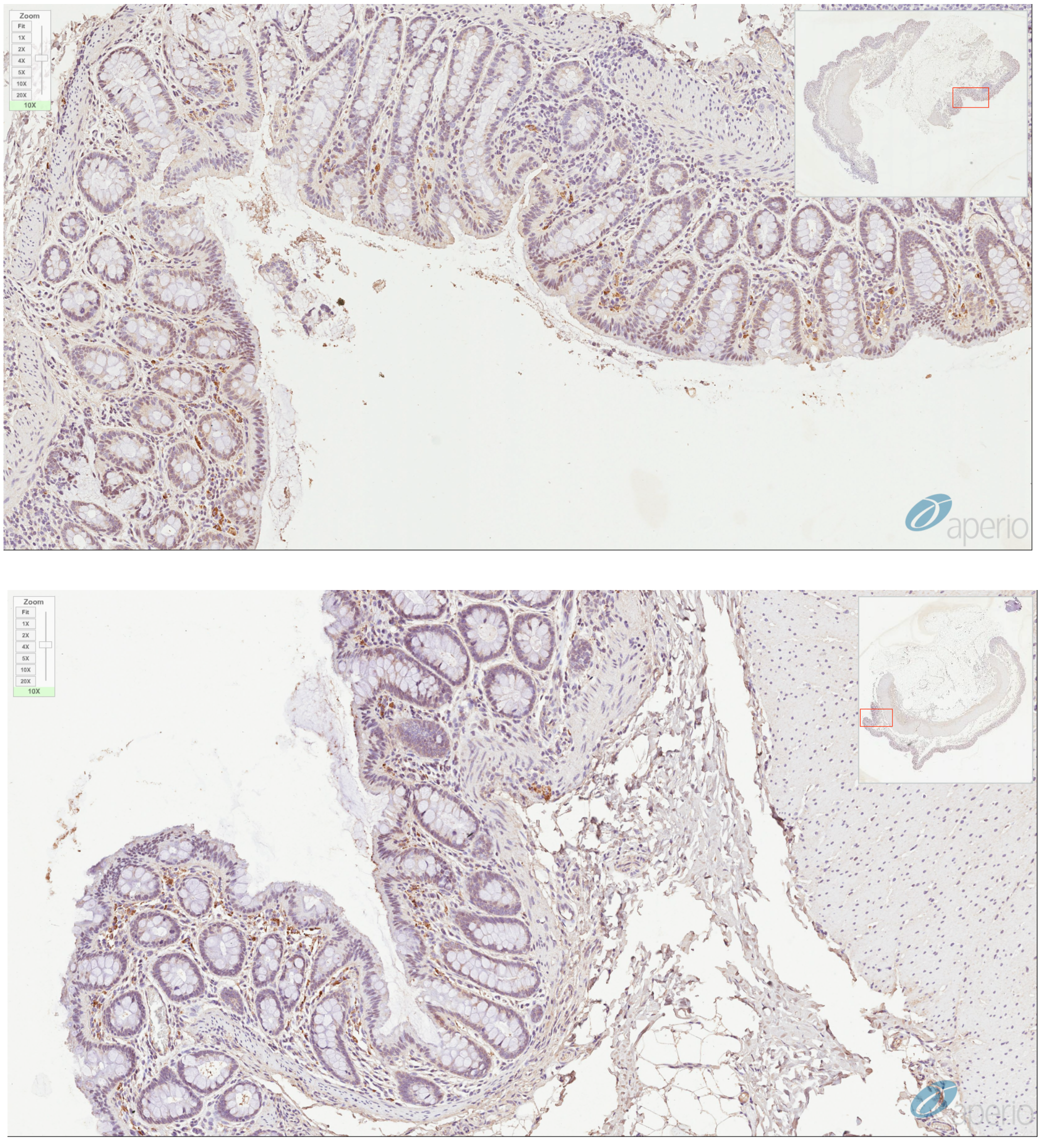

Supplementary figure 5. Immunohistochemistry of fibronectin in AGMs day 1 post SIV inoculation.

Supplementary figure 6 - AGM Day 3

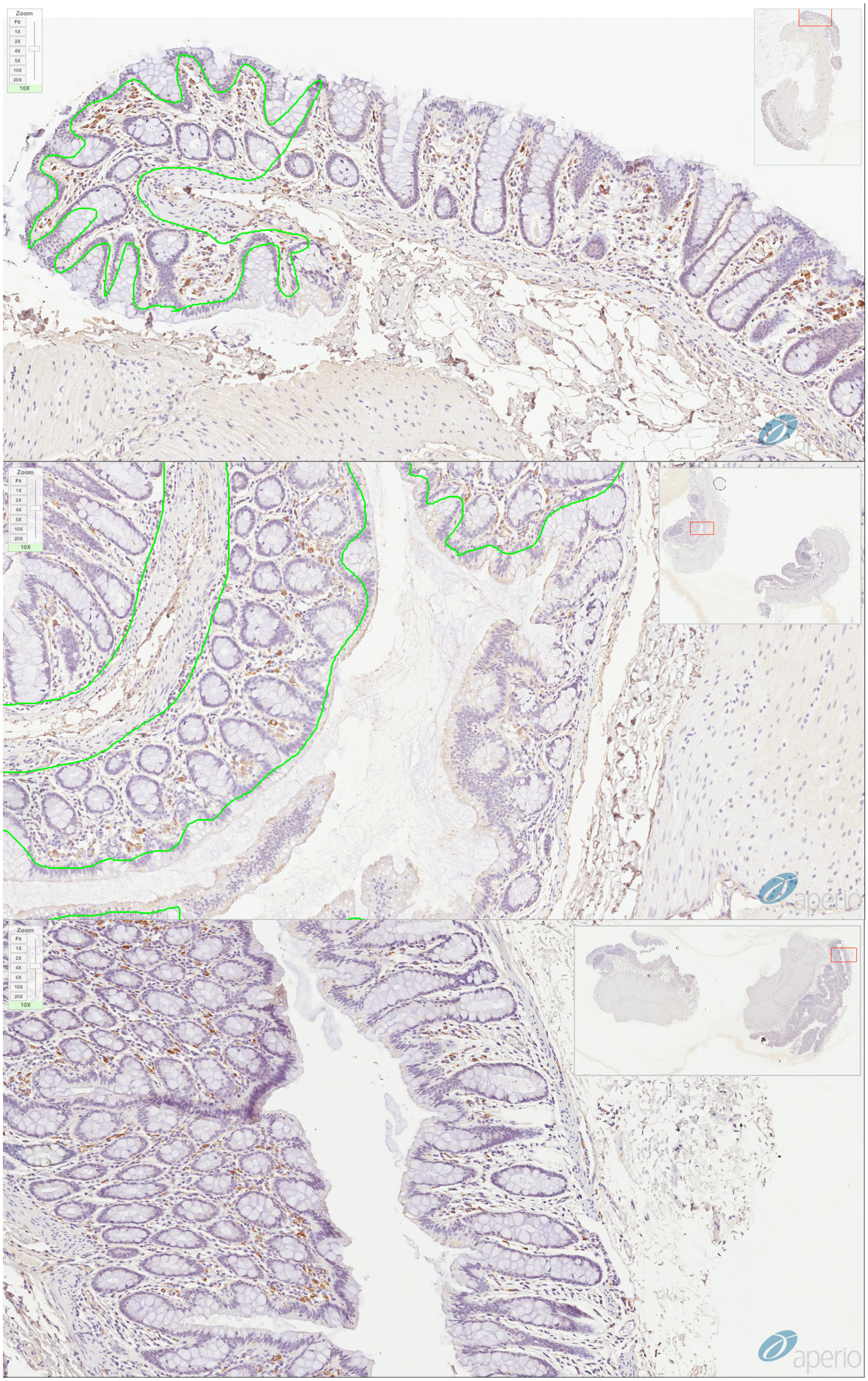

Supplementary figure 6. Immunohistochemistry of fibronectin in AGMs day 3 post SIV inoculation.

Supplementary figure 7 - RM Baseline

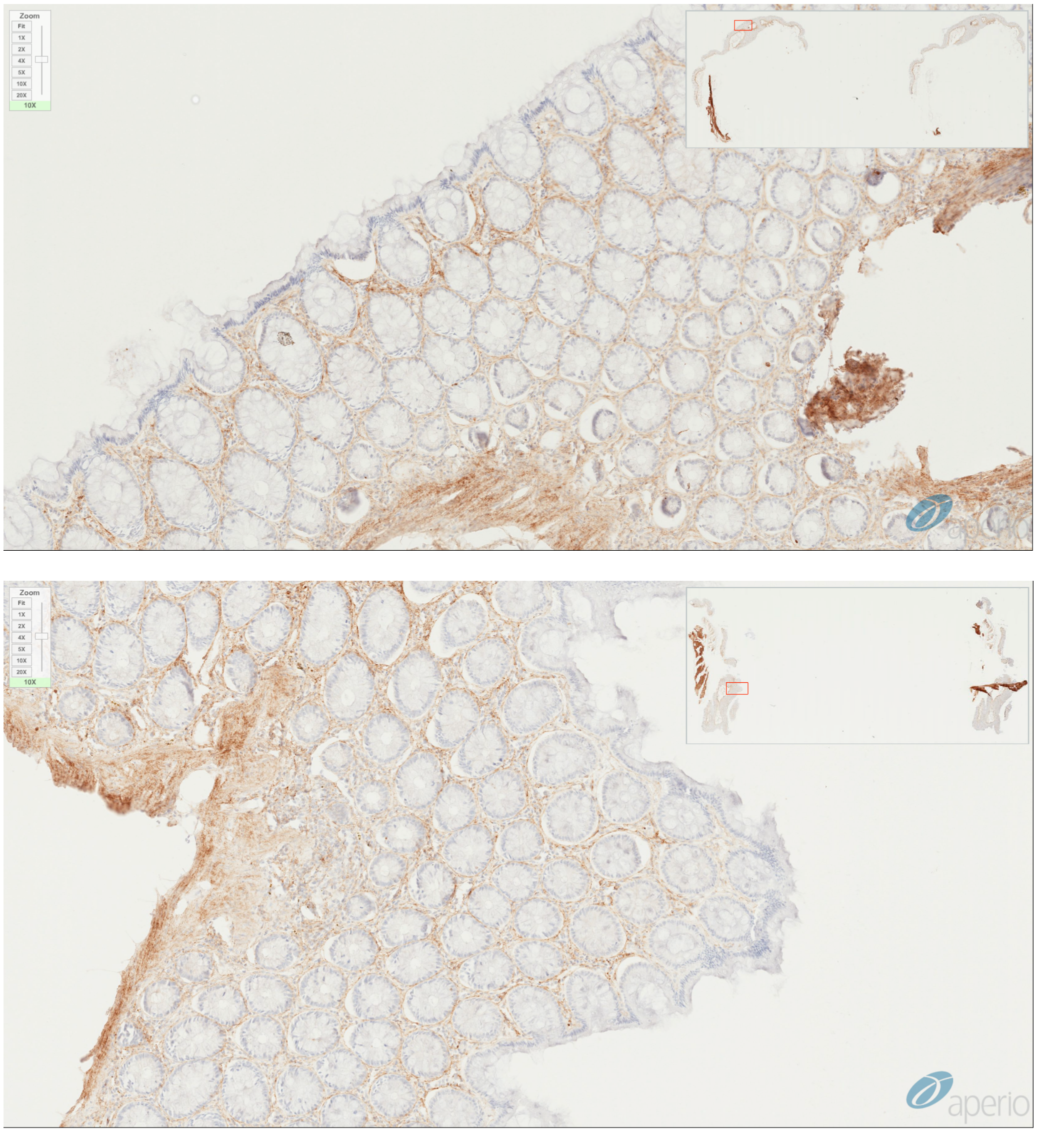

Supplementary figure 7. Immunohistochemistry of fibronectin in SIV uninfected RMs.

Supplementary figure 8 - RM Day 1

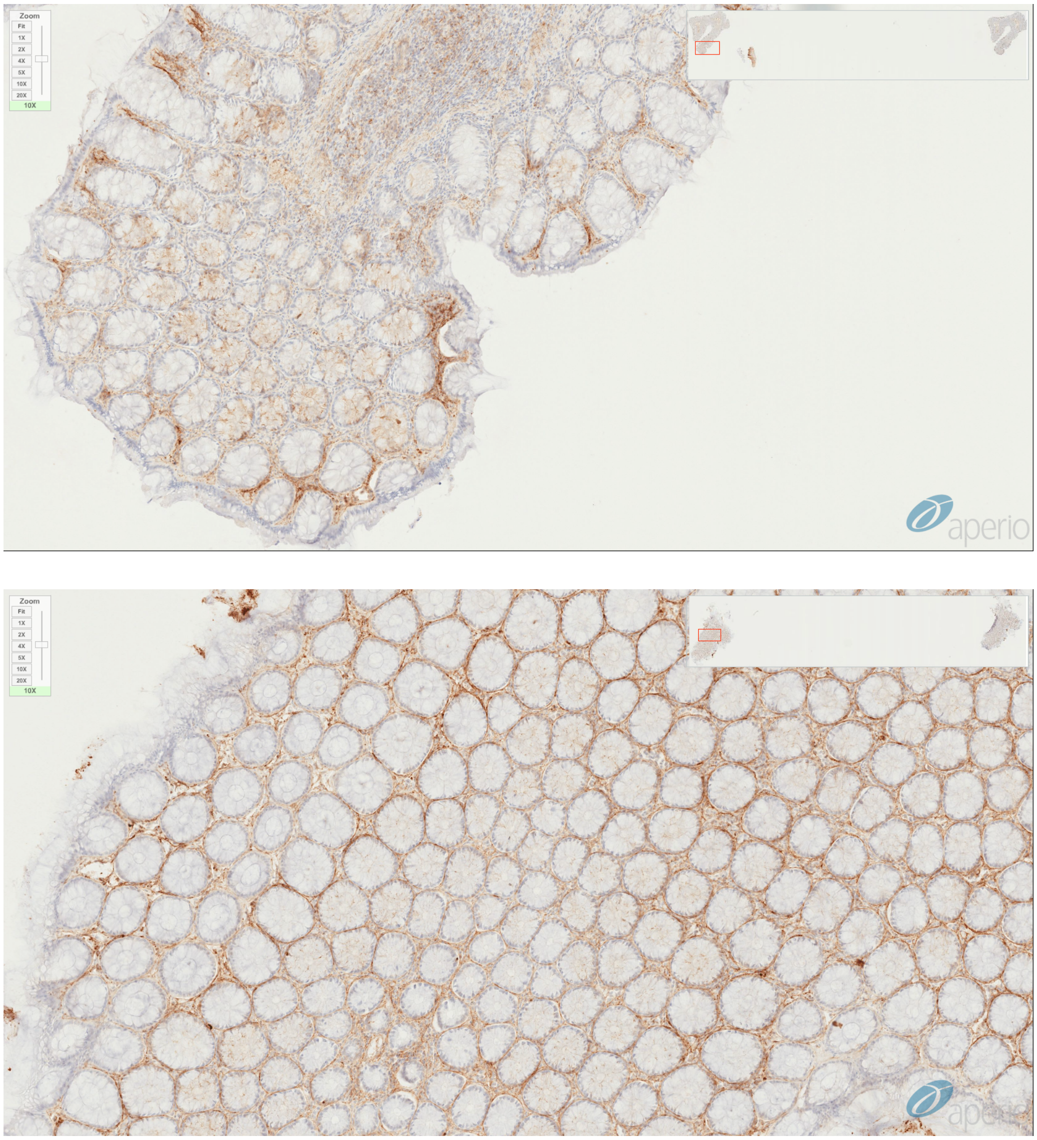

Supplementary figure 8. Immunohistochemistry of fibronectin in RMs day 1 post SIV inoculation.

Supplementary figure 9 - RM Day 3

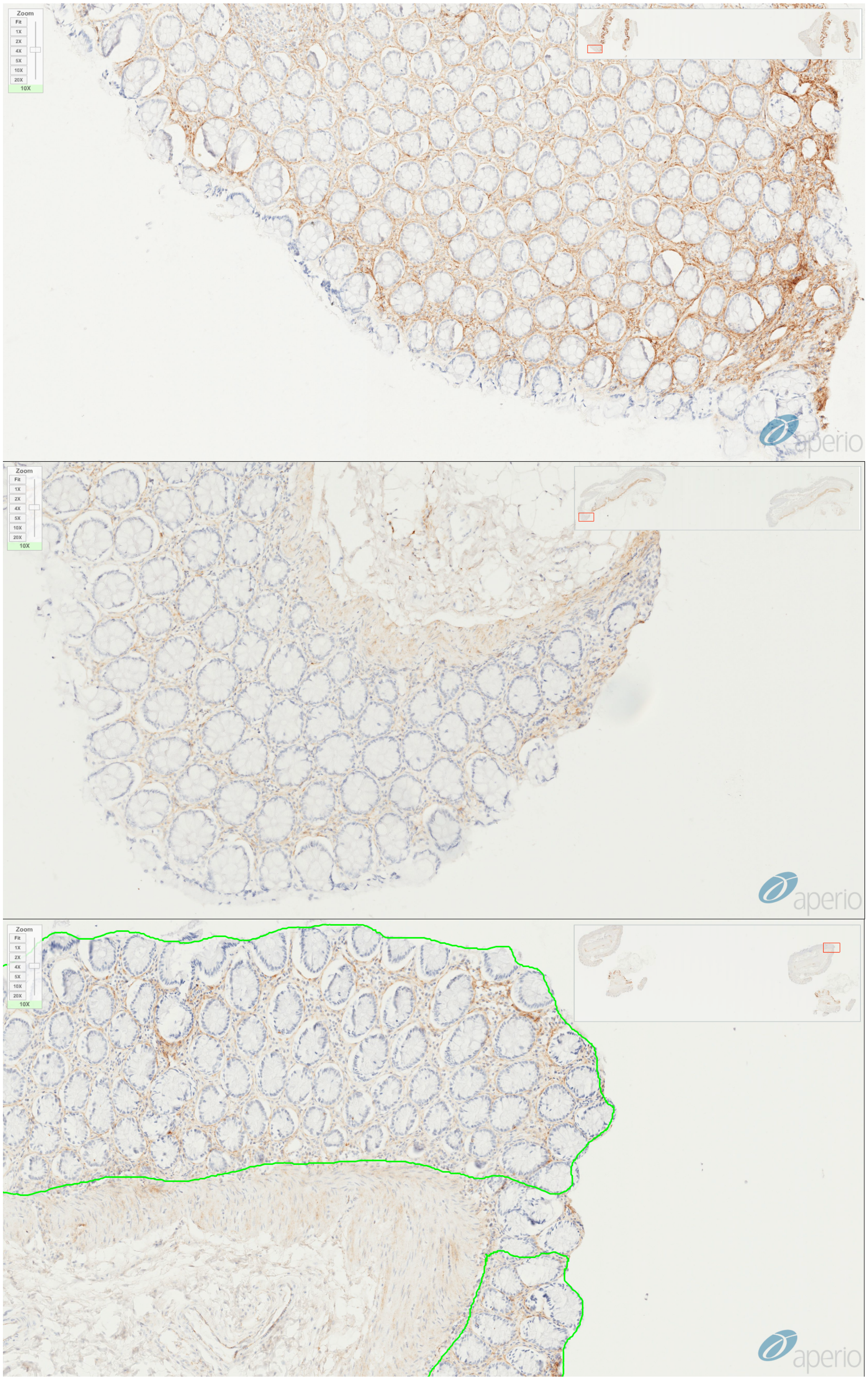

Supplementary figure 9. Immunohistochemistry of fibronectin in RMs day 3 post SIV inoculation.

Supplementary figure 10 - African Green Monkeys

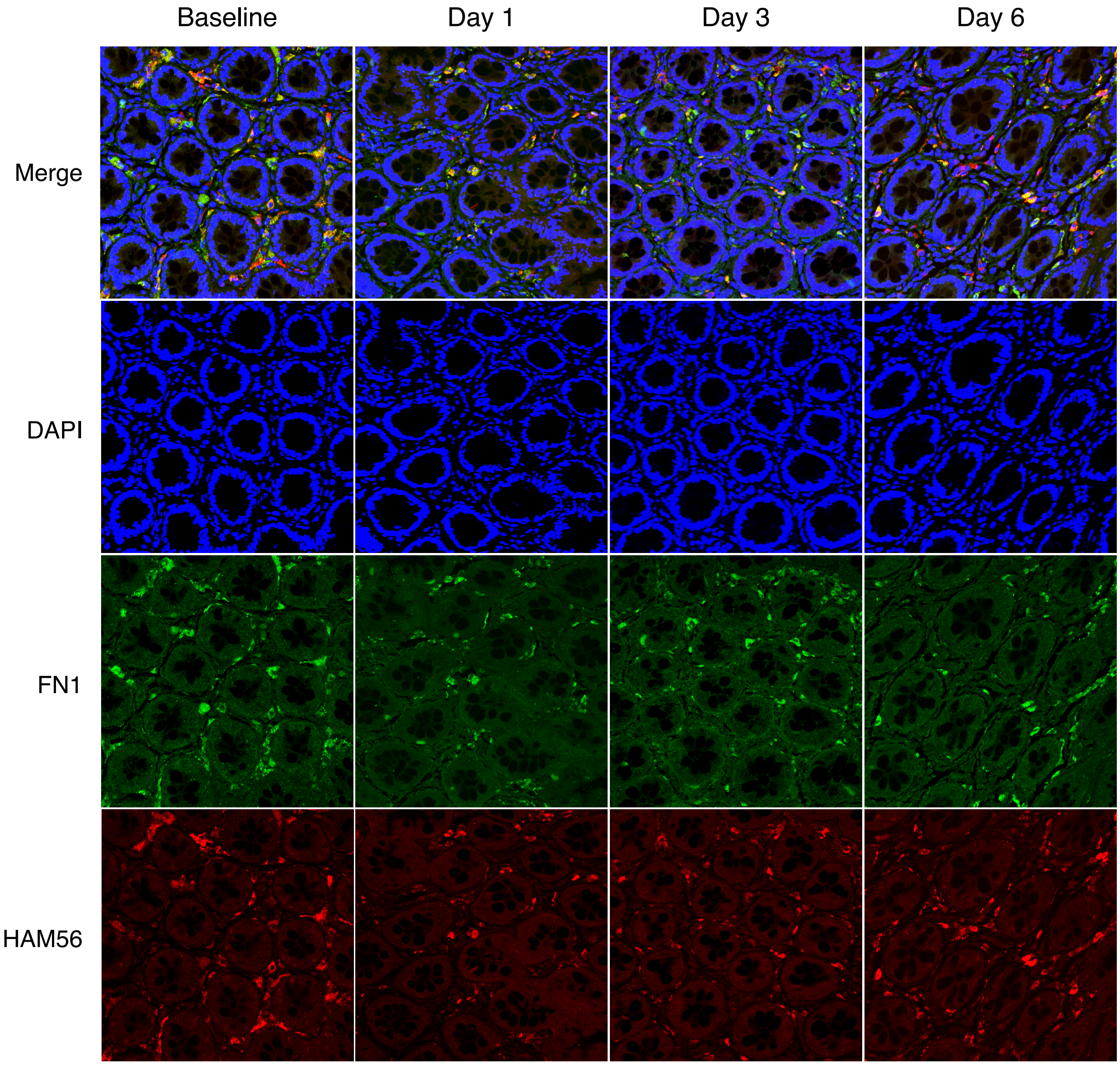

Supplementary figure 10. RGB channels for immunofluorescent staining for HAM56/FN in rectal lamina propria. Rectum from AGMs were doubled stained for HAM56 (red) and FN (green), with a DAPI stain (blue) to visualize nuclei. Colocalization, if any, appears as yellow. From top to bottom are shown the composite image and then the DAPI, FN and HAM56 color channels. From left to right, representative images are shown from 0-6 days post infetion. All images are at 200X magnification and were captured with an Olympus Fluoview 1000 Confocal Microscope housed at the Center for Biologic Imaging, Pittsburgh, PA. Each image represents a maximum intensity projection form a z-stack of 11-28 images at 1.78 μm per step. The resolution of every image is 0.321 μm/pixel. Maximum intensity projections were created with NIS Elements 5.20.00 (https://www.lim.cz/). All image editing was performed using FIJI version 2.0 (https://fiji.sc/). Editing includes adjustment of color channel brightness for clarity and application of the built-in FIJI Despeckle median filter to reduce noise.

Supplementary figure 11 - Rhesus Macaques

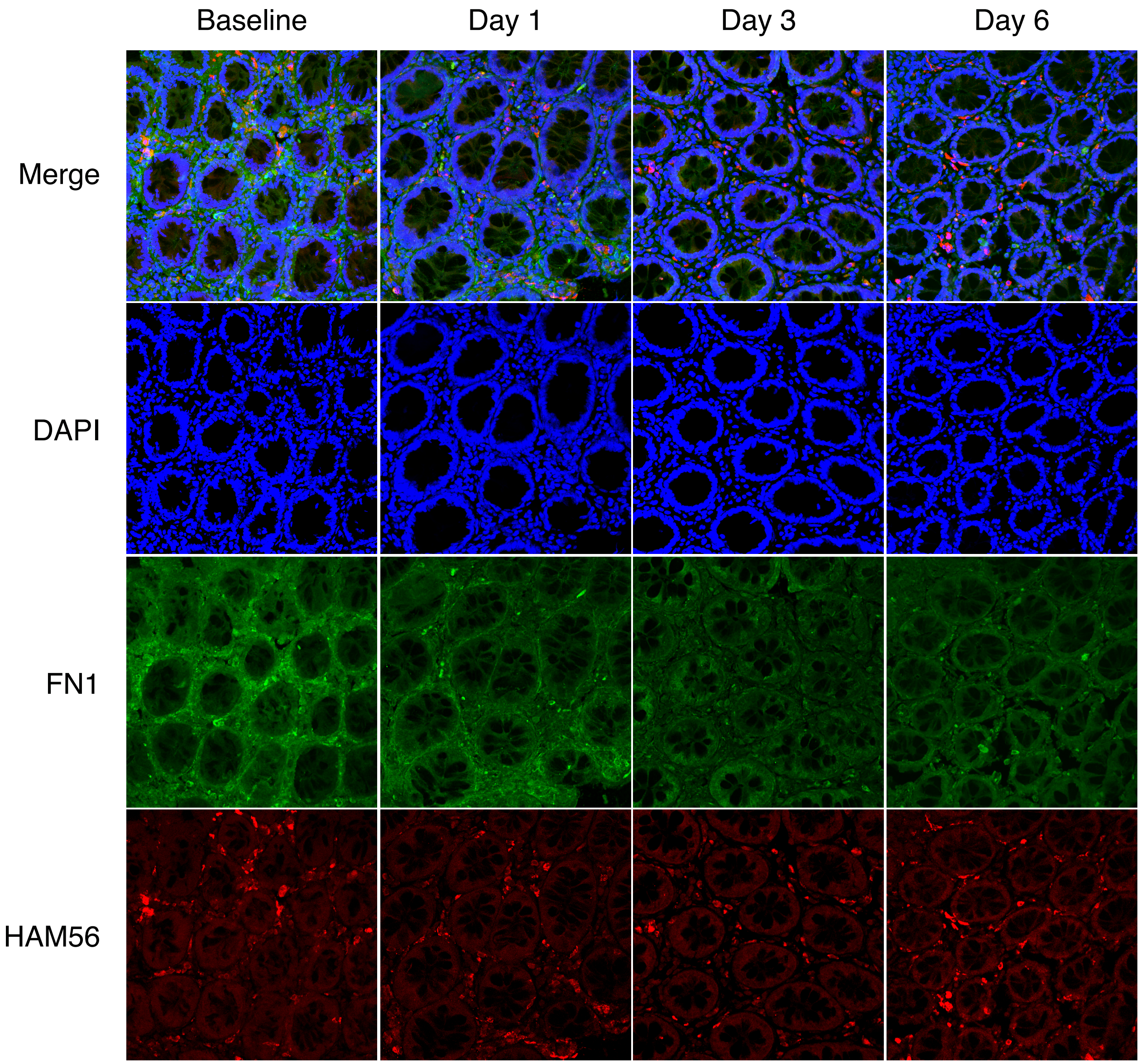

Supplementary figure 11. RGB channels for immunofluorescent staining for HAM56/FN in rectal lamina propria. Rectum from RMs were doubled stained for HAM56 (red) and FN (green), with a DAPI stain (blue) to visualize nuclei. Colocalization, if any, appears as yellow. From top to bottom are shown the composite image and then the DAPI, FN and HAM56 color channels. From left to right, representative images are shown from 0-6 days post infetion. All images are at 200X magnification and were captured with an Olympus Fluoview 1000 Confocal Microscope housed at the Center for Biologic Imaging, Pittsburgh, PA. Each image represents a maximum intensity projection form a z-stack of 11-28 images at 1.78 µm per step. The resolution of every image is 0.321 µm/pixel. Maximum intensity projections were created with NIS Elements 5.20.00 (https://www.lim.cz/). All image editing was performed using FIJI version 2.0 (https://fiji.sc/). Editing includes adjustment of color channel brightness for clarity and application of the built-in FIJI Despeckle median filter to reduce noise.

Supplementary figure 12

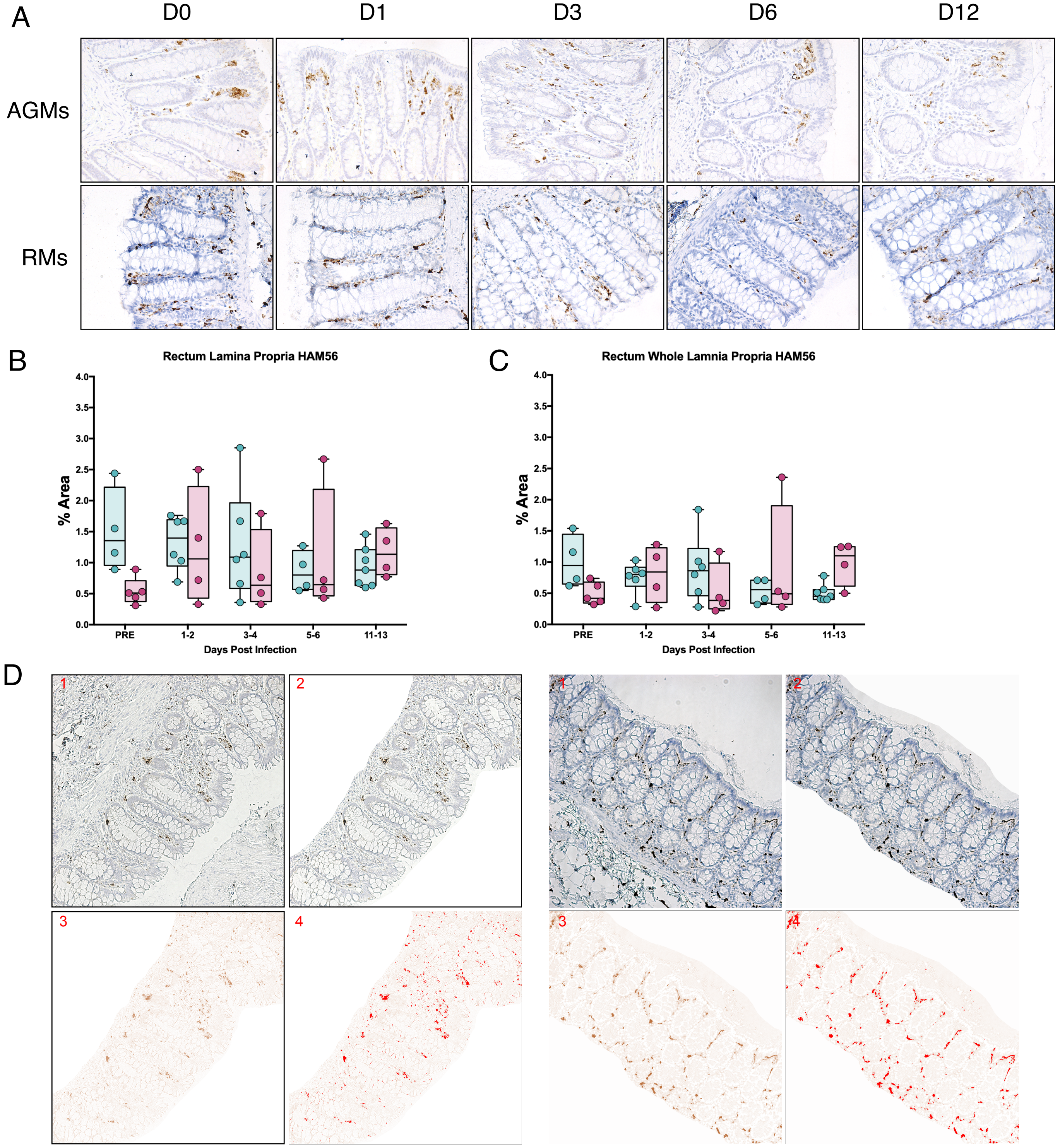

Supplementary figure 12. Immunohistochemistry & quantification of HAM56+ cells in the rectum lamina propria. DAB-based IHC for HAM56 in the rectums of AGMs and RMs, from 0-12 days post infection (A). In all images, positive DAB signal is shown in brown, with the remaining tissue counterstained blue. (B) Image based quantitative analysis was performed to measure the total positive DAB signal as percent area in the rectal lamina propria of AGMs (teal) and RMs (pink). For each animal, a total of 9-12 individual images of the lamina propria were quantified and averaged. Selection of image regions was randomized to minimize bias. (C) However, given the variable structure and cell distribution within the lamina propria, the initial quantification was verified by performing additional imaging of the entire lamina propria from the underlying muscularis mucosa to the epithelium, when possible. For each animal, 5 of these images of the whole lamina propria were quantified and averaged. (D) The strategy to isolate the lamina propria for quantification is shown for the AGMs (left) and RMs (right). For each raw image (1), the luminal space and all surrounding tissue were manually excluded from the image (2), color deconvolution was used to separate the DAB coloration (3), a threshold for color intensity was manually set to isolate the DAB signal (4). All image quantifications and editing were performed using FIJI version 2.0 (https://fiji.sc/). All images were captured at either 200X or 100X (for whole lamina propria) magnification using a Zeiss AxioImager M1 bright-field microscope equipped with an AxioCam MRc5.

Supplementary figure 13

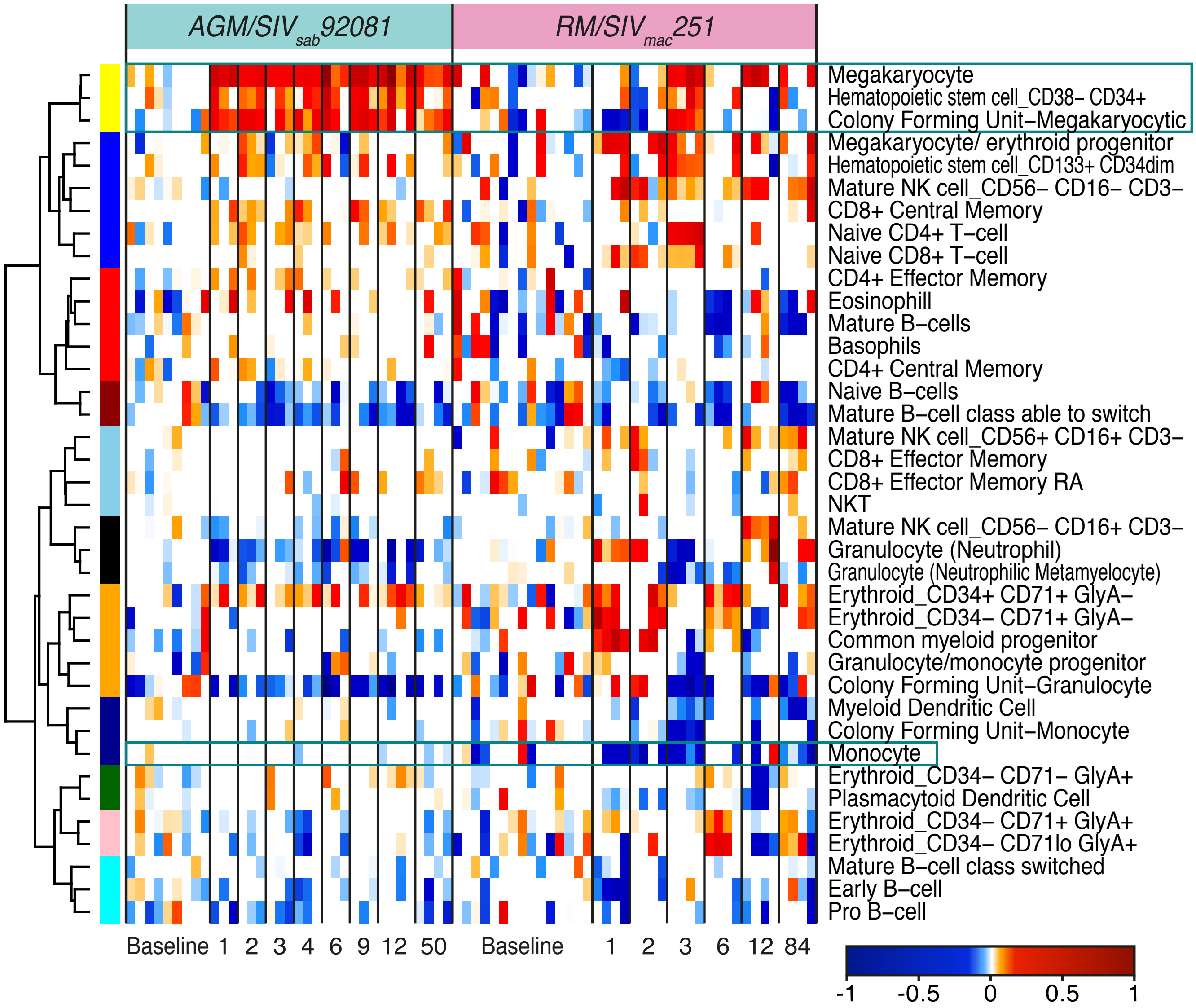

Supplementary figure 13. ImmQuant computational cell type deconvolution. Cell types are clustered using Pearson correlation and Ward D2 clustering.

Supplementary figure 14

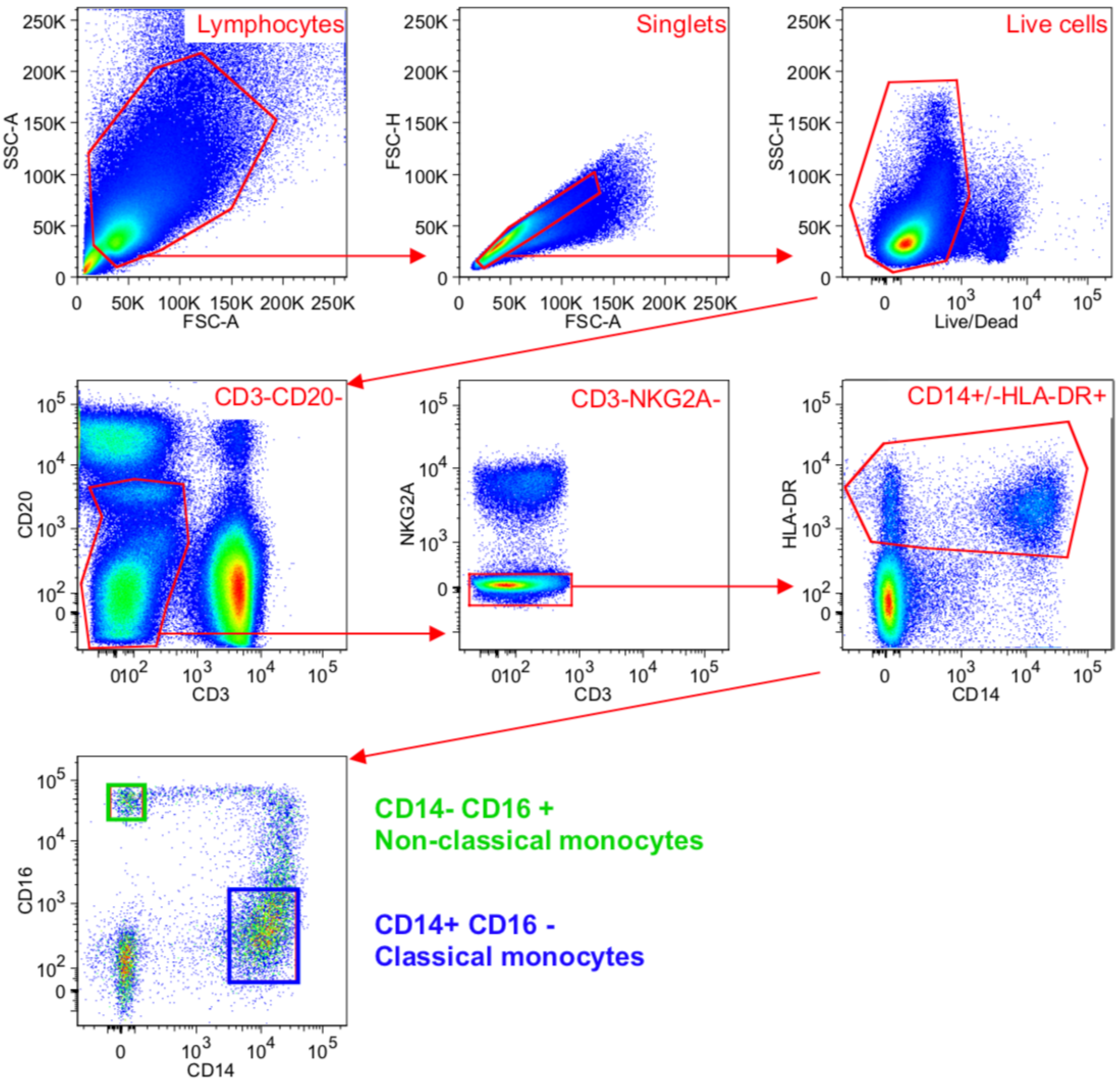

Target Protein	Fluorochrome	Clone	Manufacturer	Dilution
CD3	Alexa fluor 700	SP34-2	BD Bioscience	Manufacturer recommended volume
CD20	Brilliant violet 650	2H7	Biolegend	
NKG2A	APC	Z199	Beckman Coulter	
HLA-DR	PE-Cy7	G46-6	BD Bioscience	
CD14	ECD	RMO52	Beckman Coulter	
CD16	Brilliant Violet 421	3G8	Biolegend	
Live/Dead	AmCyan/Aqua	-	Invitrogen	1:60

Supplementary figure 14. FACS gating strategy for sorting of classical and non-classicl monocytes. Briefly, peripheral blood mononuclear cells were isolated from rhesus macaque and African green monkey whole blood by density gradient centrifugation and stained with antibodies for viability and against CD3, CD20, NKG2A, CD14, HLA-DR adn CD16 before sorting using FACS ARIA II flow cytometer.
