# Supplementary material for: Macrophage-associated wound healing contributes to African green monkey SIV pathogenesis control
Source: Nat Commun. 2019 Nov 8;10:5101. doi: 10.1038/s41467-019-12987-9 (PMC6841668; doi:10.1038/s41467-019-12987-9)
Supplement: Supplementary file 3 — Description of Additional Supplementary Files [file 41467_2019_12987_MOESM3_ESM.docx]

**Description of Supplementary Files**

**File Name:** Supplementary Data 1

**Description:** Tables detailing results from bioinformatic analyses. 1. Differential expression (DE) analyses form each time point in African green monkeys and rhesus macaques. 2. Gene Ontology Biological Process enrichment analysis for each DE list included in (1). 3. Genes included in each module of the Acute SIV Co-expression Network and Gene Ontology Biological Process enrichment analysis of each module.
