# Supplementary material for: Macrophage-associated wound healing contributes to African green monkey SIV pathogenesis control
Source: Nat Commun. 2019 Nov 8;10:5101. doi: 10.1038/s41467-019-12987-9 (PMC6841668; doi:10.1038/s41467-019-12987-9)
Supplement: Supplementary file 5 — Reporting Summary [file 41467_2019_12987_MOESM5_ESM.pdf]

nature research

Corresponding author(s): Michael Gale, Jr

Last updated by author(s): Oct 1, 2019

# Reporting Summary

Nature Research wishes to improve the reproducibility of the work that we publish. This form provides structure for consistency and transparency in reporting. For further information on Nature Research policies, see Authors & Referees and the Editorial Policy Checklist .

## Statistics

For all statistical analyses, confirm that the following items are present in the figure legend, table legend, main text, or Methods section.

| n/a | Confirmed | |
|---|---|---|
| ☐ | ☒ | The exact sample size (*n*) for each experimental group/condition, given as a discrete number and unit of measurement |
| ☐ | ☒ | A statement on whether measurements were taken from distinct samples or whether the same sample was measured repeatedly |
| ☐ | ☒ | The statistical test(s) used AND whether they are one- or two-sided<br>*Only common tests should be described solely by name; describe more complex techniques in the Methods section.* |
| ☐ | ☒ | A description of all covariates tested |
| ☐ | ☒ | A description of any assumptions or corrections, such as tests of normality and adjustment for multiple comparisons |
| ☐ | ☒ | A full description of the statistical parameters including central tendency (e.g. means) or other basic estimates (e.g. regression coefficient) AND variation (e.g. standard deviation) or associated estimates of uncertainty (e.g. confidence intervals) |
| ☐ | ☒ | For null hypothesis testing, the test statistic (e.g. *F*, *t*, *r*) with confidence intervals, effect sizes, degrees of freedom and *P* value noted<br>*Give P values as exact values whenever suitable.* |
| ☐ | ☒ | For Bayesian analysis, information on the choice of priors and Markov chain Monte Carlo settings |
| ☐ | ☒ | For hierarchical and complex designs, identification of the appropriate level for tests and full reporting of outcomes |
| ☐ | ☒ | Estimates of effect sizes (e.g. Cohen's *d*, Pearson's *r*), indicating how they were calculated |

*Our web collection on statistics for biologists contains articles on many of the points above.*

## Software and code

Policy information about availability of computer code

| | |
|---|---|
| Data collection | No software was used for data collection. |
| Data analysis | Output from the Genome Analyzer was processed using Illumina Analysis Pipeline (version 2.8). Ribosomal mapping was done using BowTie. Mapping to primate and viral genomes were determined using TopHat. Mapped read counts were assigned to exons using HT-Seq. Normalization was performed using the R library EdgeR.<br>Most statistical analyses in this study were carried out in the R environment (version 3.2.0). Significant analyses were performed using the libraries igraph (network construction and analysis), GOsim (Gene Ontology enrichment and similarity analysis), edgeR (differential expression analysis), WGCNA (correlation metrics), gplots (heatmap generation).<br>Some functional enrichment analyses were performed in Ingenuity Pathway Analysis and network visualization in Cytoscape. |

For manuscripts utilizing custom algorithms or software that are central to the research but not yet described in published literature, software must be made available to editors/reviewers. We strongly encourage code deposition in a community repository (e.g. GitHub). See the Nature Research guidelines for submitting code & software for further information.

## Data

Policy information about availability of data

All manuscripts must include a data availability statement. This statement should provide the following information, where applicable:

- Accession codes, unique identifiers, or web links for publicly available datasets
- A list of figures that have associated raw data
- A description of any restrictions on data availability

The RNA sequencing data from both rectal tissues and monocytes, can be found in Gene Expression Omnibus under accession GSE111234 [https://www.ncbi.nlm.nih.gov/geo/query/acc.cgi?acc=GSE111234].

# Field-specific reporting

Please select the one below that is the best fit for your research. If you are not sure, read the appropriate sections before making your selection.

- [x] Life sciences
- [ ] Behavioural & social sciences
- [ ] Ecological, evolutionary & environmental sciences

For a reference copy of the document with all sections, see nature.com/documents/nr-reporting-summary-flat.pdf

# Life sciences study design

All studies must disclose on these points even when the disclosure is negative.

**Sample size**: Given the nature of nonhuman primate studies, sample sizes were mainly limited by ethical concerns. The number of animals sacrificed at each time point was considered the minimal required statistical analysis and for following trends over time.

**Data exclusions**: No data was excluded from this study.

**Replication**: While no replication experiments were carried out specifically for this study, the RM samples from two time points (D3 and D12) had previously been sequenced by mRNA seq (while this study used total RNA seq) showing very high reproducibility (r2 > 0.95).

**Randomization**: Animals were randomly assigned to groups, each corresponding to a timepoint.

**Blinding**: As the aim of this study entailed comparisons between different species, blinding was not possible in this case.

# Reporting for specific materials, systems and methods

We require information from authors about some types of materials, experimental systems and methods used in many studies. Here, indicate whether each material, system or method listed is relevant to your study. If you are not sure if a list item applies to your research, read the appropriate section before selecting a response.

## Materials & experimental systems

| n/a | Involved in the study | |
|---|---|---|
| [ ] | [x] | Antibodies |
| [x] | [ ] | Eukaryotic cell lines |
| [x] | [ ] | Palaeontology |
| [ ] | [x] | Animals and other organisms |
| [x] | [ ] | Human research participants |
| [x] | [ ] | Clinical data |

## Methods

| n/a | Involved in the study | |
|---|---|---|
| [x] | [ ] | ChIP-seq |
| [ ] | [x] | Flow cytometry |
| [x] | [ ] | MRI-based neuroimaging |

## Antibodies

**Antibodies used**:

Target Protein Clone Manufacturer Reference
CD3 SP34-2 BD Bioscience 551916
CD20 2H7 Biolegend 302304
NKG2A Z199 Beckman Coulter A60797
HLA-DR G46-6 BD Bioscience 556643
CD14 RMO52 Beckman Coulter IM0645U
CD16 3G8 Biolegend 302001
Live/Dead - Invitrogen L-34965
FN (IHC) Discontinued Epitomics Inc. 3776-1
HAM56 HAM56 eBioscience 14-6548-93
FN (IF) FBN11 ThermoFisher MA5-11981
Alexa Fluor 488 Polyclonal ThermoFisher A-21121
Alexa Fluor 633 Polyclonal ThermoFisher A-21046

**Validation**: Antibody clones were chosen for our panel based on their likelihood to bind equivalently to rhesus or AGM cells, using the primary literature and testing information provided by the NIH Nonhuman Primate Reagent Resource (www.nhpreagents.org). All antibodies were tested for cross-reactivity on each species using standard titration analysis of MFI data relative to isotype controls.

CD3 Reactive in AGM and RM (PMID 25903334)
CD20 Reactive in AGM and RM according to NHP reagent resource (https://www.nhpreagents.org/NHP/clonelist.aspx?ID=63).

NKG2A Reactive in both AGM and RM according to manufacturer.
HLA-DR Reactive in AGM and RM according to NHP reagent resource (https://www.nhpreagents.org/NHP/clonelist.aspx?ID=195).
CD14 Reactive in AGM (PMID: 27221549) and RM (PMID: 26540618 )
CD16 Reactive in both AGM and RM according to manufacturer
FN (IHC) Discontinued
HAM56 Reactive in AGM and RM (PMID: 17709518)
FN (IF) Reactive in AGM (PMID: 27655016) and RM (PMID: 23321668).

## Animals and other organisms

Policy information about studies involving animals; ARRIVE guidelines recommended for reporting animal research

| | |
|---|---|
| Laboratory animals | This study entailed 24 rhesus macaques (macaca mulatta) and 28 African green monkey (chlorocebus sabaeus). All animals were adult (4-11 years) males. |
| Wild animals | This study did not involve wild animals. |
| Field-collected samples | This study did not include samples collected in the field. |
| Ethics oversight | All animal experiments in this study received ethical approval by Institutional Animal Care and Use Committees (IACUCs): University of Pittsburgh IACUC for the AGM study (protocol #1008829), University of Washington IACUC for the RM study (protocol #214207) and Emory University IACUC for the monocyte study (protocol #2002173). AGMs were housed at the RIDC animal facility of the University of Pittsburgh according to regulations set forth by the Guide for the Care and Use of Laboratory Animals and the Animal Welfare Act. RMs were housed at the Washington National Primate Research Center according to guidelines approved by the University of Washington Environmental Health and Safety Committee, the Occupational Health Administration, the Primate Center Research Review Committee. |

Note that full information on the approval of the study protocol must also be provided in the manuscript.

## Flow Cytometry

### Plots

Confirm that:

- [x] The axis labels state the marker and fluorochrome used (e.g. CD4-FITC).
- [x] The axis scales are clearly visible. Include numbers along axes only for bottom left plot of group (a 'group' is an analysis of identical markers).
- [x] All plots are contour plots with outliers or pseudocolor plots.
- [x] A numerical value for number of cells or percentage (with statistics) is provided.

### Methodology

| | |
|---|---|
| Sample preparation | AGM and RM PBMCs were isolated from EDTA-treated whole blood with dilution in sterile phosphate-buffered saline (PBS) and centrifugation for 30 minutes at 1,850 RPM at 25°C in Ficoll-Paque™ (Lonza) at a 3:2 ratio. The isolated buffy coat was washed with PBS and contaminating red blood cells lysed using an ammonium-chloride-potassium lysing buffer (Lonza) for 10 minutes before washing with PBS (10 minutes, 1800 RPM, 25°C). Cells were counted using LIVE/DEAD® Aqua Dead Cell Stain Kit (Life Technologies), surface-stained with antibodies against CD14 and CD16 (Beckman Coulter) and incubated for 30 minutes at RT before washing with FACS buffer (PBS + 2% FBS). |
| Instrument | FACS ARIA II (BD Immunocytometry) |
| Software | Data was analyzed using FlowJo |
| Cell population abundance | ~50 000 monocytes were collected from each sample. |
| Gating strategy | The gating strategy is detailed in supplementary figure 14. |

- [x] Tick this box to confirm that a figure exemplifying the gating strategy is provided in the Supplementary Information.
